# Serum proteomic changes in atopic dermatitis patients treated with cyclosporine

**DOI:** 10.1371/journal.pone.0346686

**Published:** 2026-04-20

**Authors:** Jill Isabelle Olydam, Thomas Litman, Tamar Nijsten, Ilka Hoof, Witte Koopmann, Luba Milena Pardo, DirkJan Hijnen

**Affiliations:** 1 Department of Dermatology, Erasmus University Medical Center, Rotterdam, The Netherlands; 2 Research & Early Development, LEO Pharma, Ballerup, Denmark; 3 Department of Immunology and Microbiology, University of Copenhagen, Copenhagen, Denmark; 4 Computational Biology, Research and Early Development, Novo Nordisk, Måløv, Denmark; 5 Truly Labs, Lund, Sweden; 6 Department of Dermatology, Radboud University Medical Center, Nijmegen, The Netherlands; Rutgers: Rutgers The State University of New Jersey, UNITED STATES OF AMERICA

## Abstract

**Background:**

Despite significant advancements in atopic dermatitis (AD) treatment, the underlying pathogenesis remains unclear. Understanding flare and remission mechanisms is essential for understanding disease course, evaluating treatment and developing therapeutic strategies. Although biomarkers show promise for assessing disease severity, their identification, validation and clinical utility demand further research.

**Objectives:**

To investigate inflammatory changes involved in AD flares and remissions, we studied changes in expression of 368 circulating biomarkers during cyclosporine (CsA) treatment.

**Methods:**

The study included 40 AD patients experiencing a disease flare and starting CsA treatment. Serum samples were collected at baseline, after 2 and 4 weeks of treatment.

**Results:**

CsA treatment induced a rapid improvement of disease severity and reduced proteins related to Th2, Th1 and Th17 pathways. Four novel markers, FKBP1B, PDLIM7, MAP2K6 and EIF4G1 demonstrated decreased expression levels after treatment. In addition, CCL17/TARC and OX40 levels showed a strong correlation with disease severity. Interestingly, NME3, SMOC2, and GAL showed increased expression after treatment. We identified four biomarkers that may be linked to the pathogenesis of flares and remissions. Lastly, we identified OX40 as a potential biomarker for disease severity.

**Conclusion:**

We identified four novel serum biomarkers possibly related to treatment effect and we identified OX40 as a potential serum biomarker for assessing disease severity. These findings enhance understanding of AD pathogenesis and offer tools for monitoring therapeutic response and disease severity.

## Introduction

Despite significant advancements in the treatment of atopic dermatitis (AD), the underlying pathogenesis remains largely elusive. AD is a chronic inflammatory skin disease characterized by recurrent flares and periods of remission [1,2]. Characterization of the underlying mechanism of flare and remission in the pathogenesis of AD is crucial for understanding the disease course, evaluating treatment efficacy, and developing targeted therapeutic strategies. Biomarkers have emerged as promising tools to objectively assess disease severity and activity in AD, providing valuable insights into disease pathogenesis [3–9]. However, identification and validation of reliable biomarkers for predicting treatment response and assessing disease severity in AD is an ongoing research endeavor and requires further studies to establish their clinical utility.

Until the recent introduction of biologics and JAK inhibitors for treatment of moderate-to-severe AD, CsA was the only systemic immunosuppressant registered for the treatment of atopic dermatitis in most European countries. CsA is a calcineurin inhibitor that acts by inhibiting T-cell proliferation and reducing IL-2 production, thereby decreasing inflammation in a variety of T-cell mediated diseases [10,11]. Despite its inferior side effect profile compared to the available biologics, CsA has demonstrated rapid onset of action and effectiveness in most AD patients [12–15]. Especially in young adult AD patients without co-morbidities or a history of malignancies, CsA remains an effective therapeutic option. However, patients requiring long-term systemic treatment may require transitioning to targeted therapies [16]. In several European countries, including The Netherlands, patients are still required to undergo treatment with a conventional systemic agent such as CsA before becoming eligible for targeted therapies.

Previous studies that investigated biomarker levels during CsA treatment have provided valuable insights related to treatment response and disease severity [17]. Hijnen et al. observed a correlation between serum TARC levels and clinical improvement, while Thijs et al. demonstrated a combination of biomarkers (p-EASI) as a predictor of disease severity in CsA-treated patients [7,18]. Ungar et al. noted reduced levels of Th2/Th22 and Th1 markers post-CsA treatment, correlating with SCORAD improvement [4].

The aim of the current study was to further investigate the underlying inflammatory pathways involved in AD flares and remissions by analyzing changes in the expression of a large panel of 368 circulating protein biomarkers during CsA treatment. By extending the number of proteins included in the analyses we aimed to enhance our understanding of AD disease mechanisms and identify potential biomarkers that can aid in disease monitoring and treatment response assessment.

## Methods

### Design and study population

40 adult patients with moderate-to-severe AD experiencing a disease flare were included in this prospective, exploratory, open-label study from 1st August 2020–30^th^ August 2022. We included patients diagnosed with AD using the Hanifin and Rajka criteria who were candidates for treatment with cyclosporine. A disease flare, as per the ETFAD guideline, is defined as ‘an acute, clinically significant worsening of signs and symptoms of AD requiring therapeutic intervention [19]. Patients were not allowed to use topical treatments (corticosteroids and calcineurin inhibitors) at least one week before enrollment. The major exclusion criteria were the use of systemic and/or biologic treatment (including phototherapy) within 4 weeks before the baseline visit and contradictions to cyclosporine treatment. More detailed information is provided in the Supplementary Material. Patients had moderate-to-severe disease defined as an EASI score ≥16 at baseline. Cyclosporine was administered twice daily at 3–5 mg/kg/day.

The study was approved by the local medical ethics committee of the Erasmus Medical Center Rotterdam (MEC-2019–0517; NL70997.078.19). All patients provided written informed consent to participate in the study.

### Samples and clinical data

Clinical data and Patient Reported Outcome Measures (PROMs) were collected at baseline and after 2 and 4 weeks of treatment. Physician-reported severity measures included Eczema Area and Severity Index (EASI:0–72) and Investigator Global Assessment (IGA:0–4) [20,21]. Patient Reported Outcome Measures (PROMs) included Numeric Rating Scale (NRS:0–10) peak pruritus during the past 7 days and Patient Oriented Eczema Measure (POEM: 0–28) [22,23]. Patients who achieved EASI-75 after 4 weeks of CsA treatment were defined as ‘good responders’, while patients who did not achieve EASI-75 but did achieve EASI-50 were defined as ‘responders’. No formal sample size calculations were performed given the descriptive and exploratory nature of this study. Previous studies using similar approaches have successfully included 40–60 patients and found statistically significant changes on protein and gene expression levels (PMID: 30919407, Möbus et al, JACI 2021 PMID: 32615169). A study design including 40 patients treated with cyclosporine was deemed sufficient to characterize AD flare and remission and explore molecular changes.

### Safety assessments

Safety evaluations included physical examination, recording of vital signs and laboratory examinations (including complete blood count and renal function tests) at screening/baseline and 4 weeks. Adverse events (AE), and the AE severity (categorized as mild, moderate or severe), relation to and influence on treatment were recorded at each visit. A serious AE was defined as an event that resulted in death, was life-threatening, required (prolonging of) hospitalization or resulted in persistent or significant disability.

### Blood protein quantification

Aliquoted serum samples were stored at −80 °C until further processing. Serum samples were analysed in one batch using Olink (Uppsala, Sweden) Proseek multiplex 384 inflammation assay, a proximity extension assay (PEA) with oligonucleotide-labelled antibody probe pairs [24]. The panel was one of the most extensive biomarker panels available at the time. Relative normalized protein expression (NPX) values (arbitrary log2 scale) of individual proteins can be compared across samples. Quality control (QC) was undertaken as per Olink’s standard protocol. Internal controls were added to each sample for plate and sample QC.

### Statistical analysis

Summary descriptive statistics of main demographic and clinical variables were presented as median (interquartile range) for continuous variables and as proportions for categorical variables. Clinical outcomes were analysed using the Wilcoxon signed-rank test (nonparametric, numerical outcomes) and the Fisher’s exact test (2 by 2 categorical outcomes). P-values lower than 0.05 were considered statistically significant. Analyses were performed using IBM SPSS Statistics version 25.0. Missing data were minimal and excluded from specific analyses to maintain consistency in longitudinal comparisons. Sensitivity analyses confirmed that missing data did not impact the overall patterns or correlations between biomarkers and clinical outcomes.

### Multiplex assays

Quality control of the proteomics data was performed using R (version 4.1.2) and the OlinkAnalyze package. Three assays (GBP2, HLA-E, NFASC) showed assay warnings for 11 subjects, and one sample showed QC warnings for 80 assays. These measurements were still included in the downstream data analysis. Descriptive statistics of the multiplex data are presented in the Supplementary Material S3 Table.

Statistical analysis and visualization of the filtered multiplex data was conducted with Qlucore Omics Explorer v.3.9.9 (Qlucore AB, Lund Sweden), including principal component analysis (PCA), heatmaps, unsupervised hierarchical clustering and paired t-tests. Serum expression of a panel of 368 proteins implicated in inflammatory pathways (O-link inflammation panel) was measured before and after 2 and 4 weeks of CsA treatment. Proteins, fold-changes (FCHs) >1.5 and adjusted p-values <0.05 (false discovery rate) were considered differentially expressed proteins (DEPs). Further, to take into account the protein expression changes at week 2 and week 4 simultaneously, protein-based linear models were fitted using limma with subject ID specified as a blocking factor to account for repeated measurements across visits. Visit-specific contrasts were applied to the fitted models, and empirical Bayes moderation was used to stabilize residual variance estimates across proteins. Differential expression was assessed using moderated t-statistics with p-values adjusted by the Benjamini–Hochberg false discovery rate. DEP were considered significant with an adjusted-p-value<=0.05

To evaluate for the relationship between the DEP and disease severity we used rank regression analyses. In Addition, we used limma package to calculate the correlation between proteins and EASI score, using standardized protein expression data by z-scoring protein levels. Next, we visualized the relationship between the most significant proteins and EASI score at the individual patient level using spaghetti plots.

Furthermore, we looked at the accuracy of the biomarkers in predicting the treatment response by fitting logistic regression models and AUC curves. To do this, we fitted different models, namely: 1) Logistic regression using EASI75 as the outcome and the significant proteins (from the rank correlation analysis and then calculated the ROC per protein. 2) We added sex, age and CCL17, the latter being a known biomarker for AD severity, as covariates to the first model. A final model including 23 markers was done to look at the overall predictive value of these proteins.

## Results

### Study population and clinical improvement

**Fig 1:**
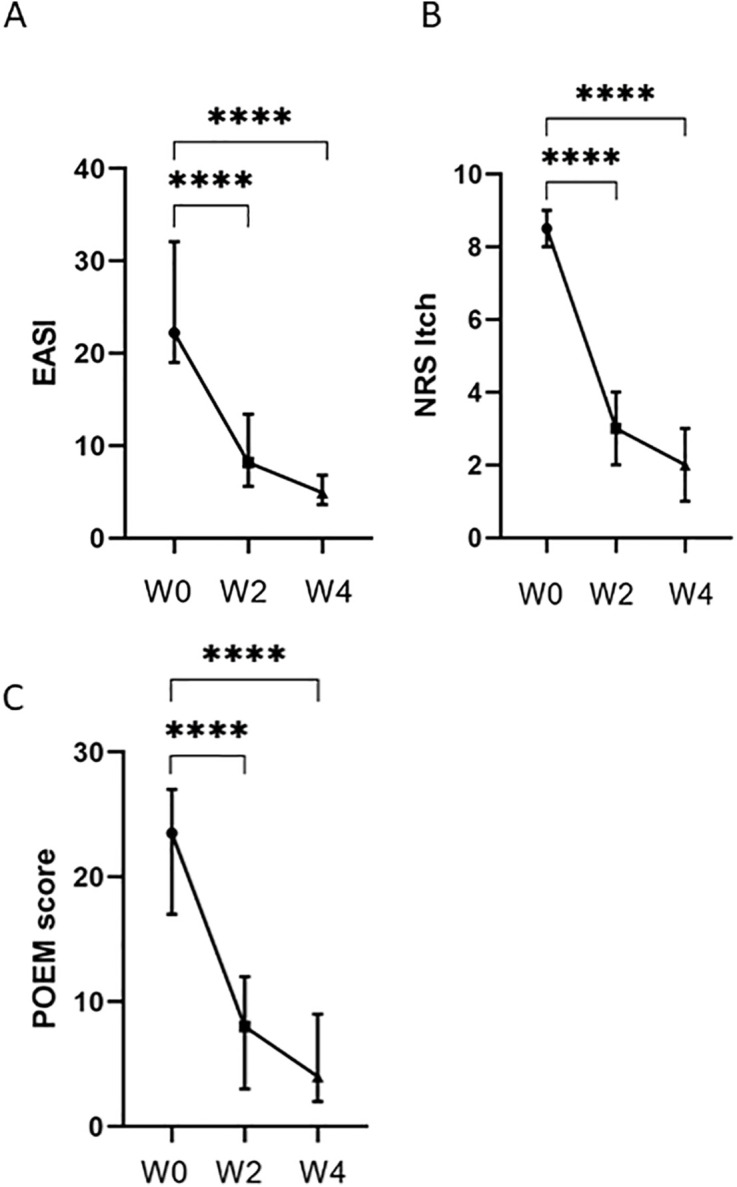
Effectiveness and patient-reported outcomes during 4 weeks of cyclosporine treatment. **(A)** Median EASI scores at baseline (week 0), after 2 weeks and after 4 weeks. **(B)** Median NRS itch scores at baseline (week 0), after 2 weeks and after 4 weeks. **(C)** Median POEM scores at baseline (week 0), after 2 weeks and after 4 weeks. Error bars represent the interquartile range. ****p < 0.0001.

Forty patients with moderate-to-severe AD (median age 29.5 (IQR 25.0–35.8)) experiencing a disease flare were included in this study. Most patients (n = 37) were naïve to oral immunosuppressive treatment (excluding glucocorticoids). CsA was started at 3–5 mg/kg daily in all patients. Patient characteristics are summarized in Table 1. CsA treatment led to a significant decrease of disease severity after two and four weeks of treatment. The median EASI score at baseline was 22.2 (IQR 19.0–32.1) and significantly decreased to 8.2 (IQR 5.6–13.4) at week 2 (p < 0.001), and to 4.9 (IQR 3.6–6.8) at week 4 (p < 0.001) (Fig 1). Seventeen patients (43.6%) reached IGA score of 0 or 1 at week 4. At week four, EASI-50, EASI-75 and EASI-90 were achieved by 97% (38/40), 55.8% (21/40) and 7.7% (3/40) of the patients, respectively (see Table 2 and S1 Fig). NRS-pruritus decreased from 8.5 (IQR 8.0–9.0) at baseline to 2.0 (IQR 1.0–3.0) after four weeks of treatment (p < 0.001). POEM scores significantly decreased from a median score of 23.5 (IQR 17.0–27.0) at baseline, to a median score of 4.0 (2.0–9.0) after four weeks of treatment (p < 0.001) (Table 2 and Fig 1). Fifty adverse events (AEs) were registered in 34 patients (85%), detailed information is shown in S1 Table. The most frequently reported adverse events were gastrointestinal discomfort or nausea (n = 15) and headache (n = 10).

**Table 1:** Baseline patient characteristics.

	AD patients (n = 40)
**Female sex** – no. (%)	18 (45.0)
**Age** – median (IQR)	29.5 (25.0-35.8)
**Fitzpatrick skin type** – no. (%)	
Type I	–
Type II	23 (57.5)
Type III	4 (10.0)
Type IV	10 (25.0)
Type V	1 (2.5)
Type VI	2 (5.0)
**Age of onset AD** years – no. (%)	
<2 years – no. (%) 2 – < 6 years – no. (%)	27 (67.5) 5 (12.5)
6 – < 18 years – no. (%)	5 (12.5)
≥ 18 years – no. (%)	3 (7.5)
**Atopic/allergic conditions** – no. (%)	
Asthma	17 (42.5)
Allergic (rhino)conjunctivitis	29 (72.5)
Allergic contact dermatitis	11 (27.5)
Food allergy, physician diagnosed	12 (30.0)
**Previous use of systemic therapies for AD**– no. (%)	
None	37 (92.5)
Cyclosporine A	1 (2.5)
Azathioprine	1 (2.5)
Tralokinumab (clinical trial)	1 (2.5)

Abbreviations: EASI, Eczema Area Severity Index,: IGA, Investigator global assessment

**Table 2:** Clinician- and patient-reported outcomes.

	Baseline n = 40	Week 2 n = 39	Week 4 n = 39	p-value
**EASI score,** median (IQR)	22.2 (19.0-32.1)	8.2 (5.6-13.4)	4.9 (3.6-6.8)	<0.0001*
**Delta EASI % from baseline**, median (IQR)	–	62.8 (52.0-74.6)	76.8 (69.1-87.0)	
**EASI-50**, no. (%)	–	32 (80.0)	38 (97.4)	
**EASI-75,** no. (%)	–	9 (23.1)	21 (55.8)	
**EASI-90,** no. (%)	–	0 (0)	3 (7.7)	
**Proportion of patients with EASI score ≤7**, no. (%)	–	16 (41.0)	30 (76.9)	
**IGA Score,** no. (%)				<0.0001*
0	–	–	–	
1	–	5 (12.5)	17 (43.6)	
2	1 (2.5)	25 (62.5)	20 (51.3)	
3	21 (52.5)	8 (20.0)	2 (5.1)	
4	18 (45.0)	1 (2.5)	–	
**Weekly average pruritus NRS score,** median (IQR)	8.5 (8.0-9.0)	3.0 (2.0-4.0)	2.0 (1.0-3.0)	<0.0001*
**Proportion of patients with NRS score ≤4**, no. (%)	–	29 (74.4)	34 (87.2)	
**POEM score,** median (IQR)	23.5 (17.0-27.0)	8.0 (3.0-12.0)	4.0 (2.0-9.0)	<0.0001*
**Proportion of patients with POEM score ≤7**, no. (%)	–	18.0 (46.2)	27 (71.1)	

Abbreviations: EASI, Eczema Area Severity Index,: IGA, Investigator global assessment; POEM, Patient Oriented Eczema Measure. * p-value for both week 0 vs 2 and week 0 vs 4

### Expression of serum proteins during cyclosporine treatment

**Fig 2:**
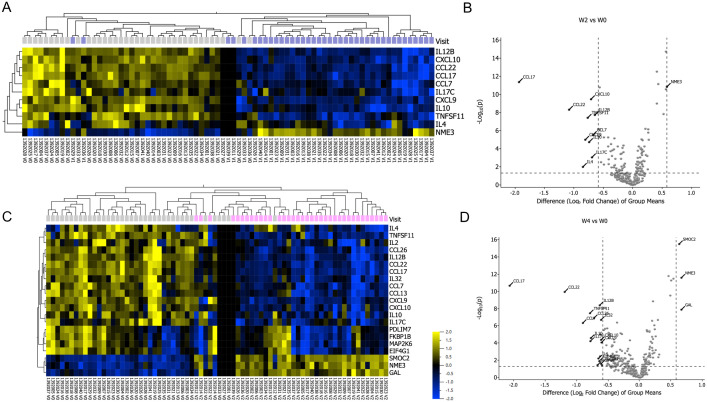
Proteomic changes after 2 and 4 weeks of CsA treatment. **(A)** Heatmap of differentially expressed proteins (DEPs) (fold change>1.5, p < 0.05, q < 0.05) after 2 weeks of CsA treatment, with markers grouped by unsupervised hierarchical clustering based on the 14 DEPs. Yellow color denotes higher and blue color denotes lower mean expression levels. **(B)** Volcano plot of DEPs after 2 weeks of treatment, the x-axis represents the value of log2-FC and the y-axis represents the mean value of –log10 (p-value). **(C)** Heatmap of DEPs (fold change>1.5, p < 0.05, q < 0.05) after 4 weeks of CsA treatment, with markers grouped by unsupervised hierarchical clustering based on the 21 DEPs. **(D)** Volcano plot of DEPs after 4 weeks of treatment, the x-axis represents the value of log2 fold change and the y-axis represents the –log10 (p-value).

Serum expression levels of 368 inflammatory proteins were measured at baseline and after 2 and 4 weeks of CsA treatment. All 368 proteins were included in the analyses, including 12 proteins with less than 25% detection across samples (see supplementary material S2 Table). Using a cutoff of fold change >1.5 and p-value <0.05, q < 0.05, paired analysis, we identified 11 and 21 differentially expressed proteins (DEPs) after 2 weeks and 4 weeks of cyclosporine treatment, of which 10 and 18 significantly decreased, respectively (Fig 2). These proteins are predominantly associated with the Th2- (CCL17, CCL22, IL-4, CCL26 and CCL13) and Th1-related pathways (CXCL10, CXCL9 and IL12B). The rapid modulation of these proteins corresponds to the rapid onset of action of CsA treatment, as evidenced by the observed changes in CCL17 and CCL22 after two weeks of treatment, which further increased by week four (Fig 2). Chemokines CCL17 and CCL22 are known markers for disease severity in AD, and CCL17/TARC is currently the best performing objective biomarker [25]. The levels of Th2 markers CCL17/TARC, CCL22/MDC, and CCL7/MCP3 (neutrophil and IL-17 pathway) showed the largest decrease after 4 weeks of CsA treatment (Fig 2C, 2D). In addition, markers associated with the IL-17 pathway, including IL-17C, CCL7 and TNFSF11, were also down regulated. Interestingly, we found that NME3 demonstrated a significant upregulation after 2 weeks of CsA treatment (Fig 2A, 2B). Notably, after four weeks of CsA treatment we identified four novel proteins (FKBP1B, PDLIM7, MAP2K6 and EIF4G1) that demonstrated decreased expression levels. In contrast, three proteins, SMOC2, NME3 and GAL, showed significant upregulation after four weeks of CsA treatment (Fig 2C, 2D). Further, we used a protein-based linear model to take into account the protein expression changes at week 2 and 4 (adjusted p-value <=0.05). The differential expressed proteins at week 2 versus baseline and week 4 versus baseline were displayed using volcano plots (supplementary Fig 4 and 5, S2 Table). The proteomic changes at 2 and 4 weeks of treatment showed an overlap of 11 proteins, mainly related to the Th2 pathway. Moreover, ten proteins were only modulated after 4 weeks of CsA treatment. Changes in individual protein levels per patients during CsA treatment are shown in S2 Fig.

### Correlation of proteins with disease severity/clinical parameters

**Fig 3:**
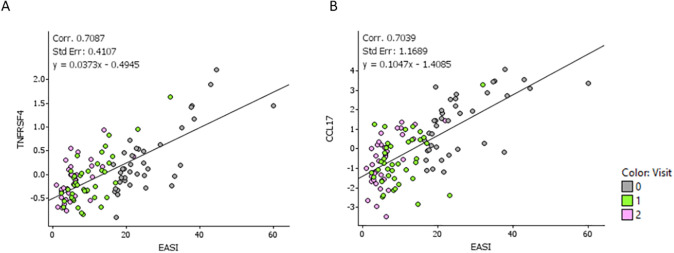
Correlations of disease severity, as measured by EASI score, with serum protein levels. **(A)** Correlation of TNFRSF4/OX40 levels and EASI score. **(B)** Correlation of CCL17/TARC and EASI score. EASI, Eczema Area and Severity Index.

Correlation analyses between protein levels and clinical scores were conducted to identify potential biomarkers that could reflect disease severity and therapeutic response. Rank regression analyses revealed significant associations between DEPs and disease severity, as measured by EASI-score. The following proteins showed the strongest correlation with EASI: CCL17 (r = 0.77), CCL22 (r = 0.72), CCL13 (r = 0.68) and CCL7 (0.64) (S3 Fig). In addition S6 Fig shows z-scores trajectories per patient per protein and EASI score. We also visualized the relationship between the most significant proteins and EASI score at individual patient level using spaghetti plots (S7 Fig) Interestingly, we also found a significant correlation for TNFRSF4/OX40 and disease severity (r = 0.70) (Fig 3).

In addition, CCL17 levels showed a significant correlation with CCL22 and OX40 levels, respectively (S8 Fig). These results confirm the potential of OX40 as a biomarker, alongside known severity markers CCL17 and CCL22, for monitoring CsA treatment response in AD patients. In addition, CCL17, CCL22 and TNFRSF4/OX40 also showed a strong correlation with patient reported outcomes POEM and NRS-pruritus. Correlations between all proteins and disease severity, as well as PROMS, can be found in supplementary material S2 Table.

### Characterization of predictive biomarkers for CsA treatment response

**Fig 4:**
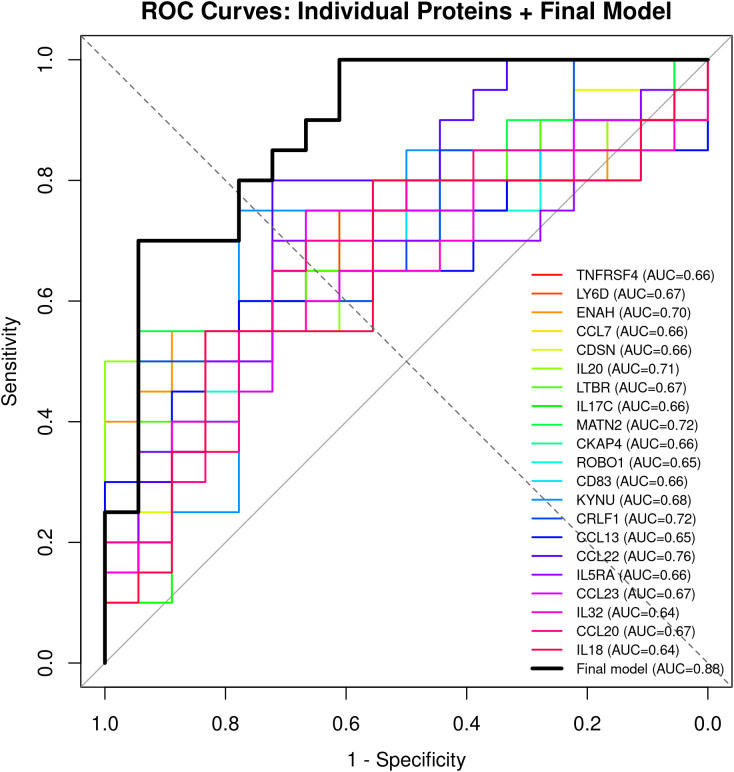
Baseline predictive biomarkers for CsA treatment response. **(A)** Heatmap of differentially expressed proteins (DEPs) (fold change <1.5, p < 0.05, q = 0.2) between>EASI 75 good responders (n = 21, red) and <EASI-75 responders (n = 17, green) at baseline, with markers grouped by unsupervised hierarchical clustering based on the 16 DEPs. Yellow color denotes higher and blue color denotes lower mean expression levels. **(B)** A volcano plot showing the differential level of proteins between>EASI 75 good responders (n = 21) and <EASI-75 responders (n = 17) at baseline. The x-axis represents the value of log2 fold change and the y-axis represents the –log-10 (p-value).

Correlations between baseline protein profiles and clinical response were investigated to identify potential biomarkers that could predict treatment response. Patients who achieved EASI-75 (n = 21) after 4 weeks of CsA treatment were defined as ‘good responders’, while patients who did not achieve EASI-75 (n = 17), but did achieve EASI-50, were defined as ‘responders’*.* Sixteen differentially expressed proteins (DEPs) were identified at baseline when comparing ‘good responders’ and ‘responders’ (t-test, p < 0.05, FC < 1.5), Fig 4). These DEPs include JUN, IRAK4 and IRAK1. A heatmap/volcano plot and unsupervised hierarchical clustering analysis based on those 16 proteins are shown in Fig 4 and demonstrate a moderate separation between the two groups. High baseline expression levels of JUN, IRAK4 and IRAK1 may be associated with a less favorable treatment response. These findings suggest that the expression levels of these proteins could potentially serve as predictive biomarkers for treatment response in a subset of AD patients. We found no significant correlation between patient demographics, including age, sex, or comorbid atopic conditions, and response to cyclosporine treatment.

**Fig 5:**
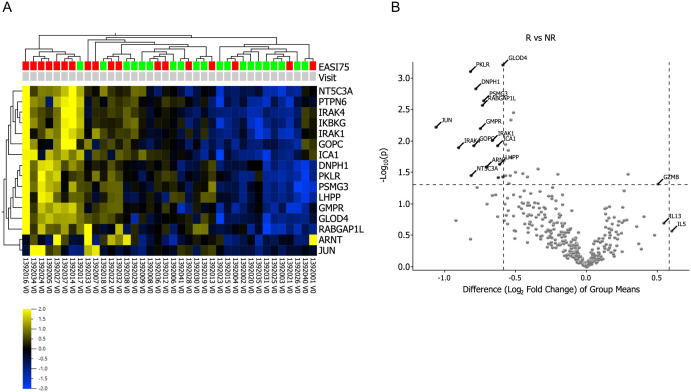
ROC model predictive biomarkers for CsA treatment response. ROC curves of a four-protein based model (black) and curves per individual protein based on odds ratio (OR) of the significant proteins predicting EASI-75 responders. Area under the ROC curve was 0.88 for the final model while individual proteins ranged from 0.6-0.7.

Additionally, we conducted a ROC curve analysis to assess the discriminatory power of the proposed biomarkers. The final model, which included the four most significant markers along with CCL17, demonstrated strong discriminatory performance (AUC = 0.88; Fig 5). Notably, TNFRSF4/OX40 demonstrated a relatively high AUC, indicating it’s potential as a predictor for treatment response. However, our model did not identify JUN, IRAK4, or IRAK1 as predictive markers.

## Discussion

In the current study we investigated the underlying inflammatory pathways of flare and remission in AD patients by analyzing changes in the protein expression of a panel of circulating biomarkers after treatment with CsA. CsA treatment was found to induce a rapid and substantial improvement in both disease severity measures and PROMS, with more than 50% of patients reaching EASI-75. Consistent with clinical remission, CsA treatment resulted in a significant reduction in the expression of circulating inflammatory biomarkers, as early as two weeks after starting treatment that continued to week 4. We observed suppression of biomarkers from multiple inflammatory pathways, particularly those related to Th2, Th1 and Th17 inflammation. The levels of Th2 markers CCL17, CCL22 and CCL7 (neutrophil and IL-17 pathway) showed the largest decrease during treatment. In addition, our study identified four new biomarkers, FKBP1B, PDLIM7, MAP2K6 and EIF4G1, which demonstrated decreased expression levels after four weeks of CsA treatment. These biomarkers may provide novel insights into the underlying mechanisms of flare and remission of disease. In addition, we identified two biomarkers (JUN and IRAK4) that may predict treatment response. Interestingly, we found that serum OX40 levels showed a strong correlation with disease severity, comparable to TARC.

Ungar et al. and Khattri et al. previously investigated proteomic and gene-expression changes during CsA treatment [4,17]. In line with our results, Ungar et al. found that CsA treatment led to a significant decrease of Th2 and Th1 related markers in serum [4]. While Ungar et al. reported the largest decrease in serum IL-22 levels, we found that serum levels of CCL17, CCL22 and CCL7 show the most significant decrease. Additionally, Ungar et al. found 9 DEPs after 12 weeks of CsA treatment, while we identified 21 DEPs already after 4 weeks of treatment. These differences may be the result of measuring a larger panel of biomarkers (n = 368) in the current study compared only 12 markers studied by Ungar et al., and a higher sensitivity of the assay used in this study.

In addition to biomarkers that are known to correlate with disease severity in AD, we identified four proteins (FKBP1B, PDLIM7, EIF4G1 and MAP2K6) that showed decreased expression levels after CsA treatment. These proteins have not been previously reported as markers associated with AD severity or to play a role in AD pathogenesis. FKBP1B plays a role in several cellular processes, including protein trafficking, signal transduction and T-cell proliferation, potentially modulating immune responses in AD [26,27]. Dubois et al. demonstrated that FKBP1B facilitates IL-15 mediated signaling in T-cells. Given the role of IL-15 in sustaining T-cell activation and memory, the observed modulation of FKBP1B expression in our study may reflect changes in adaptive immune pathways that play a role in AD pathogenesis [28]. PDLIM7, a cytoskeletal protein, participates in cell migration, adhesion and signaling pathways [29,30]. A recent study found that PDLIM7 acts as negative regulator of NF-kB signaling. NF-kB is a key transcription factor in innate immunity, critically involved in Toll-like receptor mediated activation of macrophages and dendritic cells. The observed decrease in PDLIM7 after CsA treatment may therefore indicate a downregulation of innate immune responses in AD [31]. EIF4G1, in addition to translation initiation, is implicated in mRNA regulation and interactions with cell signaling proteins [32,33]. Reduced EIF4G1 expression has been linked to enhanced inflammatory response, through upregulation of CXCL8, a chemokine that directs macrophage chemotaxis. Dysregulation of EIF4G1 may therefore contribute to the recruitment and activation [34].

MAP2K6, a kinase in the MAPK signaling pathway, is known to play a role in inflammation and immune responses [35,36]. Studies demonstrated that the activation of MAP2K6 can enhance activation of the NF-kB signaling pathway, resulting in excessive production of pro-inflammatory cytokines such as IL-6 ant TNF-a. In our study CsA treatment reduces MAP2K6 expression, suggestion that MAPK pathway activity contributes to AD pathogenesis and that modulation of this pathway may underlie part of the therapeutic effect of CsA [37].

However, the exact roles of these proteins in AD are largely unknown and need further investigation. Validation in independent AD cohorts is a necessary first step to confirm their reproducibility and added value. Moreover, in a small subset of patients these markers showed increased expression levels after treatment, confirming that AD is also heterogeneous on protein expression level. Therefore, these biomarkers may be particularly informative in specific patient subsets. Beyond mechanistic insight, evaluating biomarkers could complement classical clinical outcome measures. While measures like EASI score and IGA are widely used, they are subjective and susceptible to inter- and intra-rater variability. In contrast, serum biomarkers provide objective, quantifiable assessments of disease activity, offering reproducibility and standardization across studies.

Biomarkers predicting treatment response would be helpful for personalized treatment approaches. In the current study, we determined the relationship between baseline protein expression profiles and response to CsA treatment. While all patients showed clinical improvement, we compared serum proteomic profiles in EASI-75 responders and patients who did not reach EASI-75. JUN and IRAK4 emerged as key markers distinguishing EASI-75 responders, showing higher baseline expression levels in <EASI-75 patients. While these results are encouraging, the relatively high q-values suggest that these markers need additional validation in a larger cohort. The JUN - activator protein-1 (AP1) complex is involved in the regulation of the expression of several genes involved in immune responses [38–40], and JUN has been implicated in the downregulation of filaggrin (FLG) expression [41]. This suggests the JUN/AP-1 complex as a potential therapeutic target, as inhibition of this complex may help restoring the skin barrier function in AD.

IRAK4 is a kinase that integrates signaling downstream of receptors acting at the interface between innate and adaptive immune responses, such as Toll-like receptors, IL-1R and IL-18R [42–45]. IRAK4 inhibition has shown significant effects on key mediators, especially the Th2 cytokines IL-4 and IL-13 in various cell types, including T-cells, keratinocytes and granulocytes. Targeting IRAK4 has shown to modulate the dysregulated immune response in AD [46]. Interestingly, a recent phase I clinical trial with an IRAK4 degrading drug (KT-474) demonstrated promising results in moderate-to-severe AD patients [47]. The upregulation of JUN and IRAK4 in a subset of AD patients may indicate the presence of distinct endotypes and could potentially serve as predictive biomarkers for treatment response. Confirming these findings across different treatments would be valuable.

Interestingly, the strongest correlation with disease severity was found for TNFRSF4/OX40. OX40 is a co-stimulatory molecule expressed on T-cells [48]. The high level of circulating OX40 in patients with moderate to severe AD may reflect the high number of activated T cells in the skin of AD patients. Novel treatments targeting the OX40-OX40L pathway have shown promising results and have an acceptable safety and tolerability profile [49]. The co-stimulatory T-cell receptor OX40 and its ligand, OX40L, are thought to play an important role in the pathogenesis of AD, as their interactions are crucial for the generation of Th2 memory cells [50]. Activation of the OX40 pathway contributes to both systemic and local inflammation. Circulating CD4 + T cells show overexpression of OX40, and lesional skin T cells and dendritic cells in AD patients were found to show increased expression of OX40 and OX40L respectively [51,52]. Elsner et al. recently reported a correlation between OX40 levels and SCORAD in children, but they did not find this in adult AD patients [53]. OX40 has the capability to bind to its ligand expressed by activated antigen-presenting cells, e.g., dendritic cells, endothelial cells, macrophages and activated B-cells [50,54,55]. This interaction could contribute to the observed decrease in serum levels and the variations in correlations with disease severity. Mechanistic studies further demonstrate that blocking OX40 signaling not only reduces Th2-assocatied mediators (including IL-31, CCL11 and CCL17) but also modulates Th1 and Th17/Th22 signatures in lesional skin. These findings indicate that OX40 captures broader immune activation than TARC/CCL17 alone, making it a more comprehensive and mechanistically informative biomarker for disease activity [52,56]. Our study had several limitations. We included only patients with severe disease, experiencing a flare and requiring systemic treatment in the study. In addition, using a pre-defined inflammation biomarker panel results in a selection bias, potentially excluding interesting other markers. The findings of this study require confirmation and validation in an independent cohort.

In conclusion, rapid clinical improvement was seen within two weeks of CsA treatment, and this was associated with a significant decrease in key Th2 related circulating inflammatory proteins. After four weeks of treatment, we observed a decrease in the expression of proteins associated with Th2, Th1 and Th17 inflammation. Furthermore, we identified IRAK4 as a potential predictor for CsA treatment response. Lastly, we demonstrated a strong correlation between OX40 levels and disease severity. This indicates that OX40 not only holds promise as a therapeutic target but also emerges as a potential objective biomarker for assessing disease severity.

## Supporting information

S1 File Supplement inclusion and exclusion criteria. (DOCX)

S1 Table Adverse events. (DOCX)

S2 Table Correlations between all proteins and disease severity. (XLSX)

S1 Fig Percentage patients achieving EASI 50/75/90. (DOCX)

S2 Fig Changes in protein levels per patient. (DOCX)

S3 Fig Correlations of disease severity. (DOCX)

S4 Fig Volcano plot differential expressed proteins at week 2 versus baseline. (DOCX)

S5 Fig Volcano plot differential expressed proteins at week 4 versus baseline. (DOCX)

S6 Fig Z-score trajectories per patient for protein and EASI score. (DOCX)

S7 Fig Spaghetti plot protein expression and changes in EASI score. (DOCX)

S8 Fig Correlation between OX40 and CCL17. (DOCX)

## Abbreviations

AD: atopic dermatitis
CsA: cyclosporine
DEPs: differentially expressed proteins
EASI: Eczema Area and Severity Index
IGA: Investigator Global Assessment
IQR: interquartile range
NRS: Numeric Rating Scale
PEA: proximity extension assay
POEM: Patient Oriented Eczema Measure
PROMS: Patient Reported Outcome Measures
TARC/CCL17: thymus and activation-regulated chemokine/C-C motif chemokine ligand 17
