# Correction: Serum proteomic changes in atopic dermatitis patients treated with cyclosporine

**DOI:** 10.1371/journal.pone.0357330

**Published:** 2026-08-31

**Authors:** The PLOS One Staff

The images for Figs 4 and 5 are incorrectly switched. The image that appears as Fig 4 should be Fig 5, and the image that appears as Fig 5 should be Fig 4. The figure captions appear in the correct order. The authors have provided a corrected version of the figures here.

The publisher apologizes for the error.

**Fig 4:**
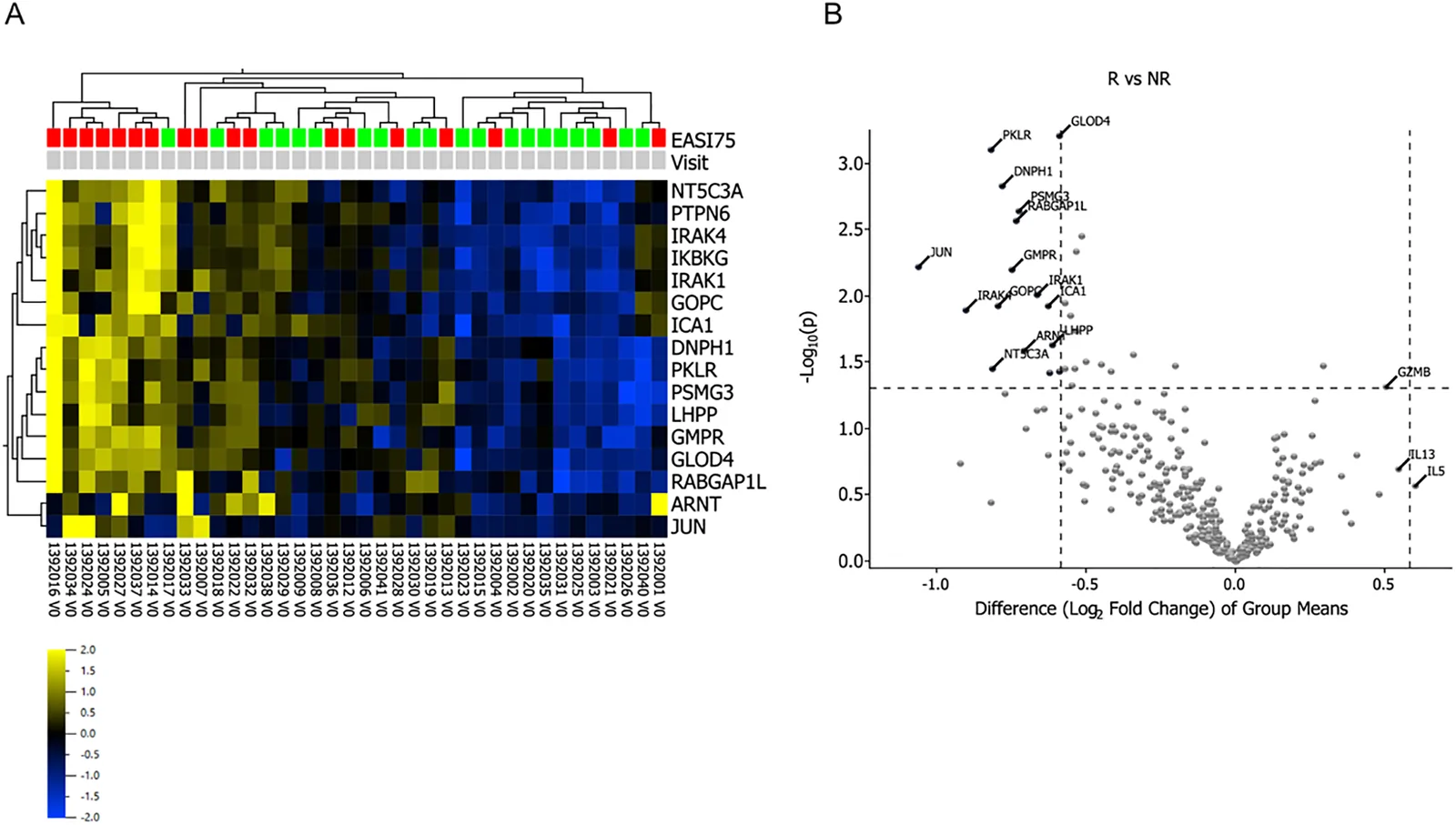
Baseline predictive biomarkers for CsA treatment response. **(A)** Heatmap of differentially expressed proteins (DEPs) (fold change <1.5, p < 0.05, q = 0.2) between>EASI 75 good responders (n = 21, red) and <EASI-75 responders (n = 17, green) at baseline, with markers grouped by unsupervised hierarchical clustering based on the 16 DEPs. Yellow color denotes higher and blue color denotes lower mean expression levels. **(B)** A volcano plot showing the differential level of proteins between>EASI 75 good responders (n = 21) and <EASI-75 responders (n = 17) at baseline. The x-axis represents the value of log2 fold change and the y-axis represents the –log-10 (p-value).

**Fig 5:**
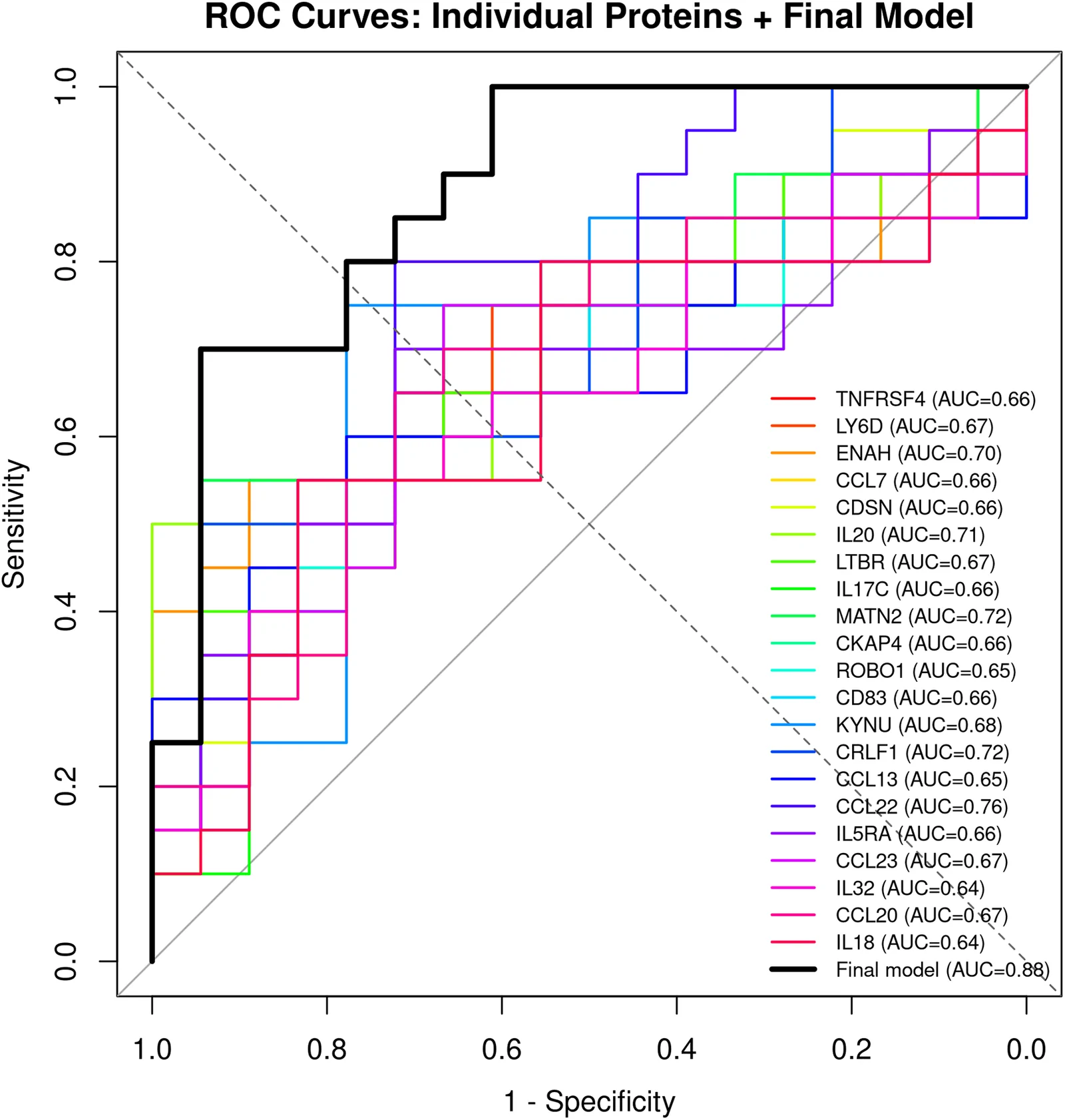
ROC model predictive biomarkers for CsA treatment response. ROC curves of a four-protein based model (black) and curves per individual protein based on odds ratio (OR) of the significant proteins predicting EASI-75 responders. Area under the ROC curve was 0.88 for the final model while individual proteins ranged from 0.6-0.7.

## References

[1] Olydam JI, Litman T, Nijsten T, Hoof I, Koopmann W, Pardo LM, et al. Serum proteomic changes in atopic dermatitis patients treated with cyclosporine. PLoS One. 2026;21(4):e0346686. doi: 10.1371/journal.pone.0346686 42008513 PMC13094968

