# Supplementary material for: Serum proteomic changes in atopic dermatitis patients treated with cyclosporine
Source: PLoS One. 2026 Apr 20;21(4):e0346686. doi: 10.1371/journal.pone.0346686 (PMC13094968; doi:10.1371/journal.pone.0346686)
Supplement: S1 File — (DOCX) [file pone.0346686.s001.docx]

Inclusion criteria and exclusion criteria

STUDY POPULATION

Population

Patients with atopic dermatitis and intention to start cyclosporine A treatment, who meet with

the in- and exclusion criteria. Patients will be recruited at the dermatology outpatient clinic of

the Erasmus University Medical Centre Rotterdam.

Inclusion criteria

In order to be eligible to participate in this study, a subject must meet all of the following

criteria:

1. Signed and dated informed consent has been obtained prior to any protocol related

procedures.

2. Diagnosis and history of chronic, moderate-to-severe AD (by the Eichenfield revised

criteria of Hanifin and Rajka [18]) for at least 3 years before the screening visit.

3. Age 18 years or above.

4. Subjects who have an AD flare at the start of the study (EASI score ≥10 at screening and

≥16 at the baseline visit).

5. An intention to start cyclosporine A treatment.

6. Willing and able to comply with the clinical study protocol.

7. Subjects able to read and understand, and willing to sign the informed consent form

Exclusion criteria

A potential subject who meets any of the following criteria will be excluded from participation

in this study:

1. Treatment with allergen immunotherapy within 6 months before the baseline visit.

2. Treatment with leukotriene inhibitors within 4 weeks before the baseline visit.

3. Treatment with systemic glucocorticosteroids within 4 weeks before the baseline visit.

4. Treatment with topical corticosteroids (TCS) or topical calcineurin inhibitors within 1 week

before the baseline visit.

5. Systemic treatment for AD with an immunosuppressive/immunomodulating substance,

e.g. cyclosporine A, mycophenolate-mofetil, interferon-γ (IFN-γ), phototherapy (narrow

band ultraviolet B [NBUVB], ultraviolet B [UVB], ultraviolet A1 [UVA1], psoralen +

ultraviolet A [PUVA]), azathioprine, or methotrexate within 4 weeks before the baseline

visit.

6. Previous treatment with biologics within 4 weeks prior to the baseline visit.

7. Receipt of live attenuated vaccines 4 weeks prior to the baseline visit. Receipt of

inactive/killed vaccinations (e.g. inactive influenza) is allowed, provided they are not

administered within 5 days before/after any study visit.

8. Subjects who have received treatment with any non-marketed drug substance (that is, an

agent which has not yet been made available for clinical use following registration) within

4 weeks prior to the baseline visit.

9. Chronic or acute infection requiring treatment with oral or intravenous antibiotics, anti

virals, anti-parasitics, anti-protozoals, or anti-fungals within 4 weeks before the screening

visit or superficial skin infections within 1 week before the baseline visit.

10. Use of a tanning booth/parlour within 4 weeks before the screening visit.

11. Current participation in any other interventional clinical trial.

12. Previous enrolment in this study.

13. Employees of the trial site or any other individuals directly involved with the planning or

conduct of the trial, or immediate family members of such individuals.
