# Supplementary material for: Serum proteomic changes in atopic dermatitis patients treated with cyclosporine
Source: PLoS One. 2026 Apr 20;21(4):e0346686. doi: 10.1371/journal.pone.0346686 (PMC13094968; doi:10.1371/journal.pone.0346686)
Supplement: S1 Table — (DOCX) [file pone.0346686.s002.docx]

Table S1. Adverse events

| **Adverse events (AE)** | **Number** |
| --- | --- |
| Subjects | 40 |
| Total no. of AE | 50 |
| Serious AE | 0 |
| Subjects with at least 1 AE, no. (%) | 34 (85) |
| Gastrointestinal discomfort/nausea, no. (%) | 15 (30.0) |
| Neuromuscular AE, no. (%)  Headache  Fatigue  Muscle cramps  Paraesthesia | 11 (22.0)  3 (6.0)  3 (6.0) 10 (20.0) |
| Hypertrichosis, no. (%) | 1 (2.0) |
| Gingival hyperplasia, no. (%) | 2 (4.0) |
| Hypertension, no. (%) | 1 (2.0) |
| Creatinine increase > 30%, no. (%) | 0 |
| Flushing, no. (%) | 3 (6.0) |
| Hair loss, no. (%) | 1 (2.0) |
