# Supplementary material for: Serum proteomic changes in atopic dermatitis patients treated with cyclosporine
Source: PLoS One. 2026 Apr 20;21(4):e0346686. doi: 10.1371/journal.pone.0346686 (PMC13094968; doi:10.1371/journal.pone.0346686)
Supplement: S1 Fig — (DOCX) [file pone.0346686.s004.docx]

Figure S1: Percentage patients achieving EASI 50/75/90


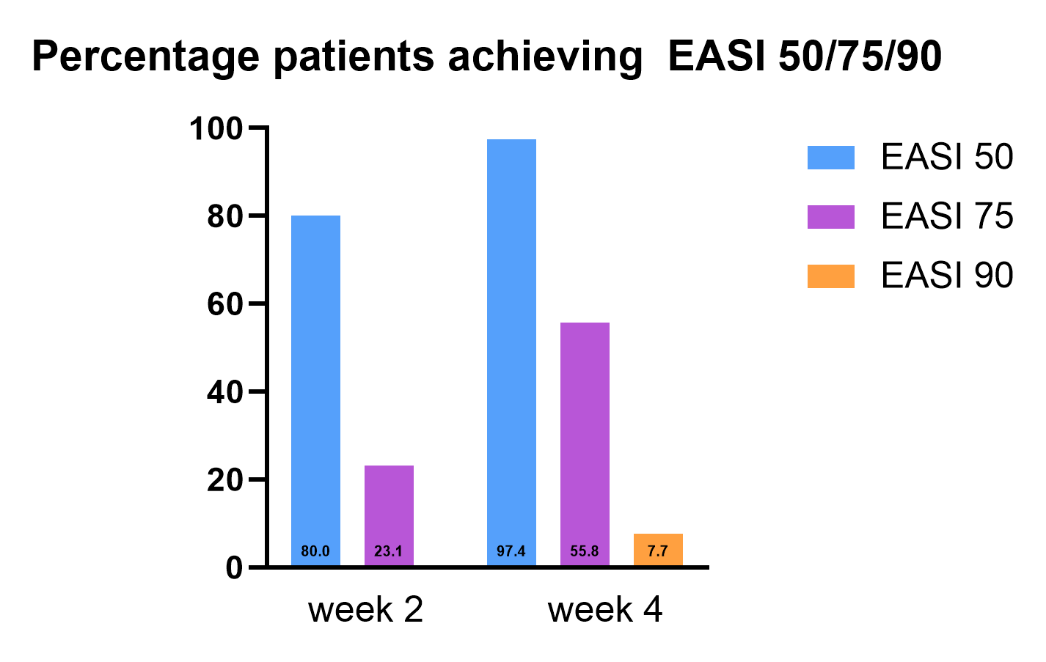


Figure S1. EASI outcomes after 2 and 4 weeks of cyclosporine treatment. Proportion of patients achieving EASI-50, EASI-75 and EASI-90 ****p<0.0001. EASI, Eczema Area Severity Index.
