# Supplementary figures and images for: Serum proteomic changes in atopic dermatitis patients treated with cyclosporine
Source: PLoS One. 2026 Apr 20;21(4):e0346686. doi: 10.1371/journal.pone.0346686 (PMC13094968; doi:10.1371/journal.pone.0346686)

Figure S2: Changes in protein levels per patient


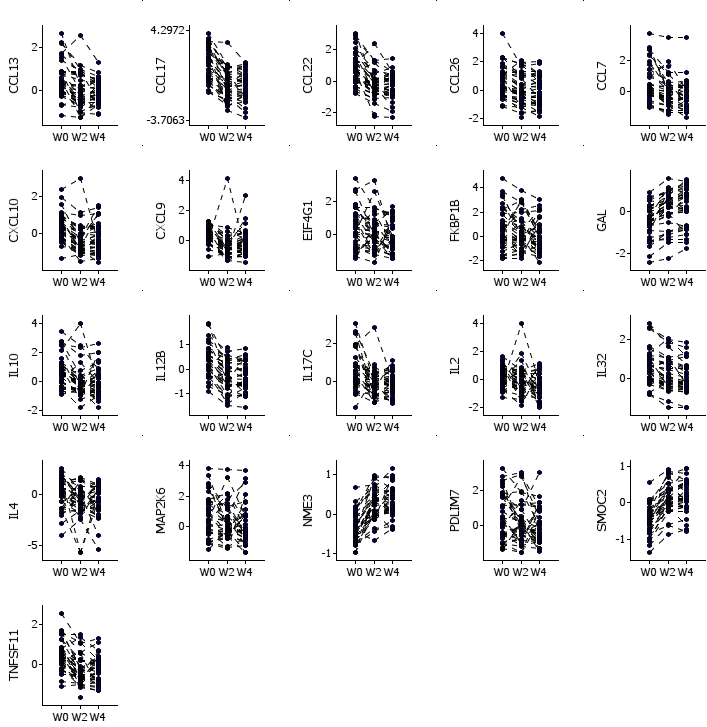

Supplement: S2 Fig — (DOCX) [file pone.0346686.s005.docx]

Figure S8 correlation between OX40 (TNFRSF4) and CCL17 (TARC)


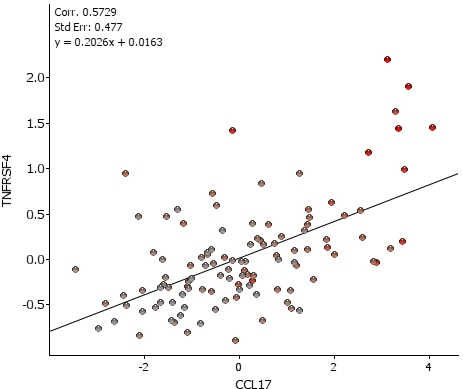

Supplement: S8 Fig — (DOCX) [file pone.0346686.s011.docx]
