# Supplementary material for: Serum proteomic changes in atopic dermatitis patients treated with cyclosporine
Source: PLoS One. 2026 Apr 20;21(4):e0346686. doi: 10.1371/journal.pone.0346686 (PMC13094968; doi:10.1371/journal.pone.0346686)

Figure S3: Correlations of disease severity, as measured by EASI score, with serum protein levels. EASI, Eczema Area and Severity Index.


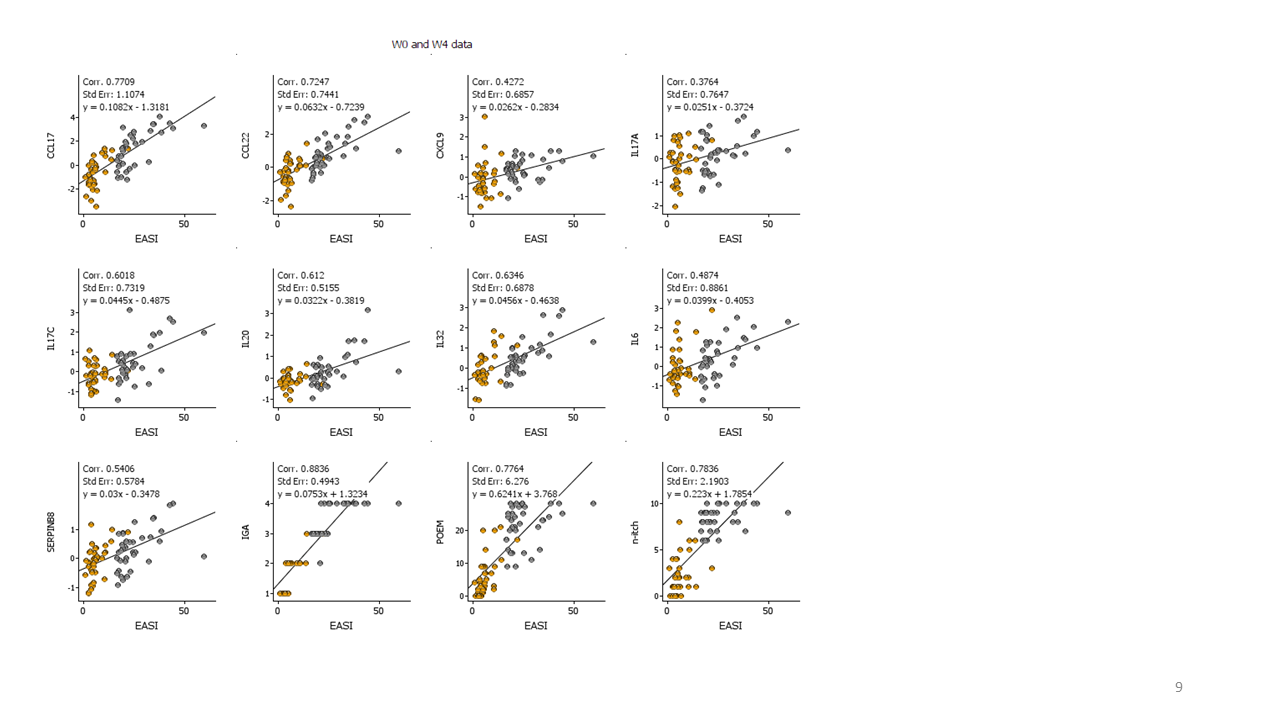

Supplement: S3 Fig — (DOCX) [file pone.0346686.s006.docx]
