# Supplementary material for: Serum proteomic changes in atopic dermatitis patients treated with cyclosporine
Source: PLoS One. 2026 Apr 20;21(4):e0346686. doi: 10.1371/journal.pone.0346686 (PMC13094968; doi:10.1371/journal.pone.0346686)
Supplement: S4 Fig — (DOCX) [file pone.0346686.s007.docx]

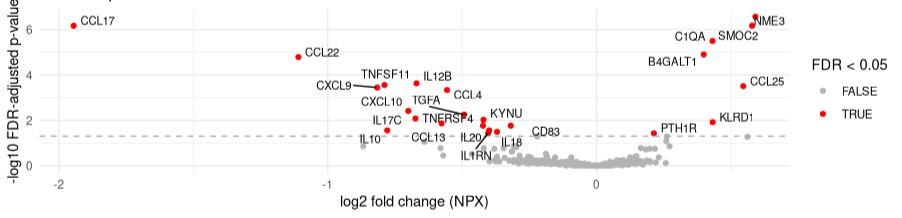


F

Figure S4 Volcano plot differential expressed proteins at week 2 versus baseline

A volcano plot of differential expressed proteins (DEPs) (adjusted p-value <=0.05) after 2 weeks of treatment.
