# Supplementary material for: Serum proteomic changes in atopic dermatitis patients treated with cyclosporine
Source: PLoS One. 2026 Apr 20;21(4):e0346686. doi: 10.1371/journal.pone.0346686 (PMC13094968; doi:10.1371/journal.pone.0346686)
Supplement: S6 Fig — (DOCX) [file pone.0346686.s009.docx]

Figure S6 z-score trajectories per patient for protein and EASI score.


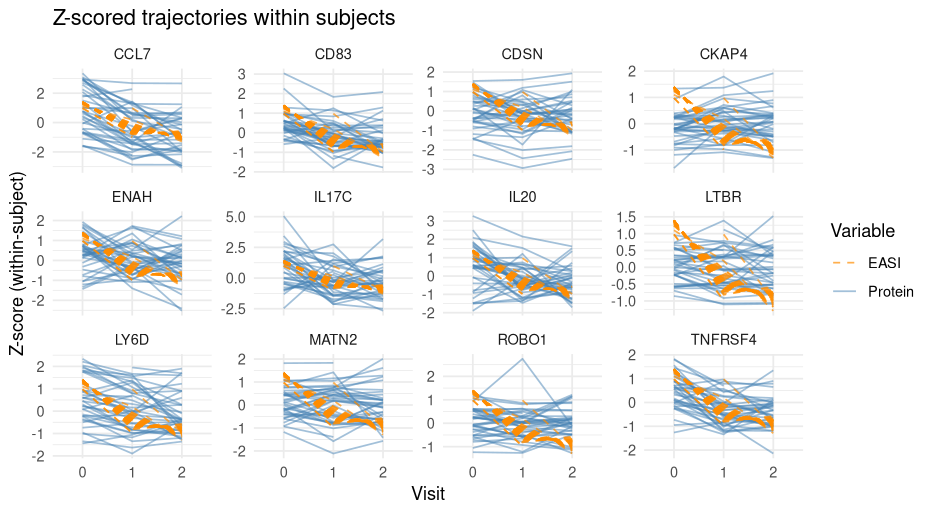


Figure S6 Z-scored trajectories per patient for proteins in relationship with EASI score per study visit. , The blue lines indicate changes in protein, the orange line indicates changes in EASI score.
