# Supplementary material for: Serum proteomic changes in atopic dermatitis patients treated with cyclosporine
Source: PLoS One. 2026 Apr 20;21(4):e0346686. doi: 10.1371/journal.pone.0346686 (PMC13094968; doi:10.1371/journal.pone.0346686)
Supplement: S7 Fig — (DOCX) [file pone.0346686.s010.docx]

Figure S7 Spaghetti plot protein expression and changes in EASI score
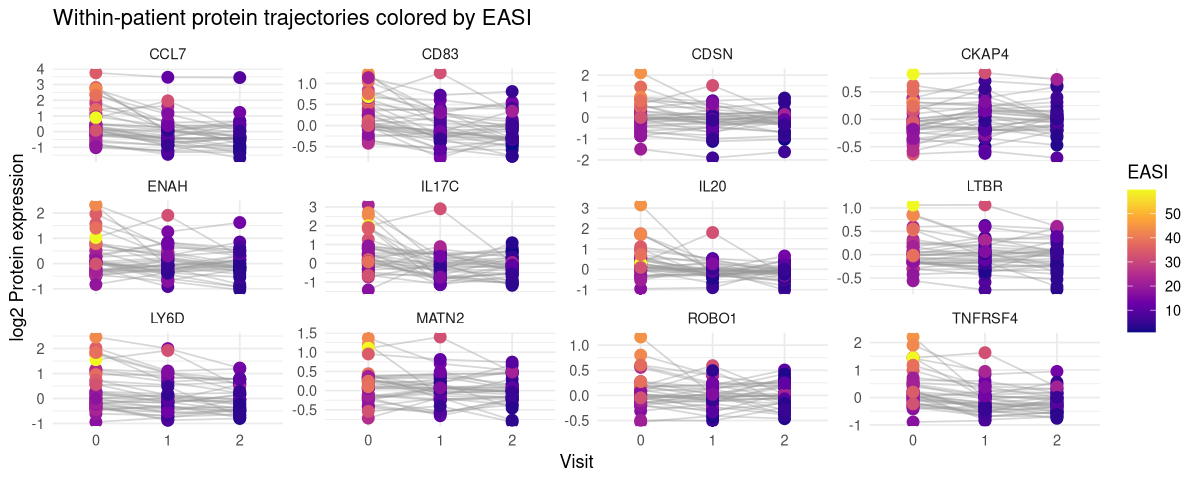


Figure S7 Protein changes (log2 fold changes) per patient in relationship with EASI score per study visit.
